# Physical symptom burden in patients with desmoid‐type fibromatosis and its impact on health‐related quality of life and healthcare use

**DOI:** 10.1002/cam4.5985

**Published:** 2023-04-29

**Authors:** Anne‐Rose W. Schut, Leanne E. de Bruin, Belle H. de Rooij, Emma Lidington, Milea J. M. Timbergen, Winette T. A. van der Graaf, Winan J. van Houdt, Johannes J. Bonenkamp, Robin L. Jones, Dirk. J. Grünhagen, Stefan Sleijfer, Spyridon Gennatas, Cornelis Verhoef, Olga Husson

**Affiliations:** ^1^ Department of Medical Oncology Erasmus MC Cancer Institute Rotterdam The Netherlands; ^2^ Department of Surgical Oncology Erasmus MC Cancer Institute Rotterdam The Netherlands; ^3^ Department of Research and Development Netherlands Comprehensive Cancer Organisation Utrecht The Netherlands; ^4^ CoRPS—Center of Research on Psychology in Somatic Diseases/Department of Medical and Clinical Psychology Tilburg University Tilburg The Netherlands; ^5^ Sarcoma Unit Royal Marsden NHS Foundation Trust London UK; ^6^ Department of Medical Oncology Netherlands Cancer Institute Amsterdam The Netherlands; ^7^ Department of Surgical Oncology Netherlands Cancer Institute Amsterdam The Netherlands; ^8^ Department of Surgical Oncology Radboud University Medical Center Nijmegen The Netherlands; ^9^ Division of Clinical Studies Institute of Cancer Research, Royal Marsden NHS Foundation Trust London UK; ^10^ Department of Medical Oncology, Guy's and St Thomas' NHS Foundation Trust London UK

**Keywords:** desmoid tumour, healthcare utilisation, health‐related quality of life, patient‐reported outcomes, rare diseases

## Abstract

**Background:**

Desmoid‐type fibromatosis (DTF) has a highly variable clinical course with varying intensity of symptoms. The objectives of this study were to identify subgroups of DTF patients based on physical symptom burden and to compare symptom burden subgroups on health‐related quality of life (HRQoL) and healthcare use (univariate and multivariate).

**Methods:**

Desmoid‐type fibromatosis patients from the United Kingdom and the Netherlands received cross‐sectional questionnaires on HRQoL (EORTC QLQ‐C30), DTF‐specific HRQoL (DTF‐QoL) and healthcare utilisation. Latent class cluster analysis was performed to identify subgroups based on patients' symptom burden using EORTC QLQ‐C30 and DTF‐QoL physical symptom items. Multivariate linear and logistic regression analyses were conducted to examine associations of symptom burden with HRQoL and healthcare utilisation, respectively.

**Results:**

Among 235 DTF patients, four symptom burden clusters were identified, with low symptom burden (24%), intermediate symptom burden‐low pain (20%), intermediate symptom burden‐high pain (25%) and high symptom burden (31%). DTF patients with high symptom burden had clinically relevant lower HRQoL scores compared to patients with low and intermediate symptom burden (*p* < 0.001) and reported more general and DTF‐related visits to their general practitioner compared to the low symptom burden cluster (*p* < 0.01). In the multivariate analyses, symptom burden was independently associated with both HRQoL and healthcare utilisation.

**Conclusions:**

This study identified four distinct subgroups of DTF patients based on their level of symptom burden, with a considerable number of patients being highly symptomatic. Knowledge of the level of symptom burden DTF patients experience can help to identify patients at risk of poorer outcomes and tailor supportive care to the individual needs of DTF patients.

## 1 INTRODUCTION

Desmoid‐type fibromatosis (DTF) is a rare, intermediate‐grade soft tissue tumour, which does not metastasize but can display locally aggressive tumour growth.^1^ It usually affects young adults and can arise in any part of the body, most commonly the abdominal wall and extremities.^2^, ^3^, ^4^ DTF has an unpredictable clinical and biological behaviour, with phases of disease progression, stabilisation or spontaneous tumour regression without any treatment.^5^, ^6^, ^7^ The unpredictable character makes DTF challenging to treat. Active surveillance (AS) is currently recommended as first line treatment, while active treatment, including systemic therapies, surgical resection and local therapies, may be considered in case of progressive and symptomatic disease.^8^

DTF patients may experience psychological distress and pain or other physical complaints caused by the tumour itself, treatment complications or toxicity.^9^, ^10^, ^11^ Symptom presentation among DTF patients, however, is highly variable, ranging from no symptoms at all to extreme pain and functional limitations, regardless of tumour behaviour or size.^7^ To better understand which patients experience low or high symptom burden, studies in cancer patients have used cluster analyses to identify subgroups with the same degree of symptom burden.^12^, ^13^, ^14^ In these studies, patients with higher symptom burden reported worse health‐related quality of life (HRQoL) outcomes. Moreover, patients with higher symptom burden tend to make more use of healthcare services.^15^, ^16^, ^17^ In DTF, the extent of symptom burden patients experience and its impact on HRQoL and healthcare use has not yet been studied. Since DTF patients constitute a heterogeneous population with different tumour locations, treatment strategies and healthcare needs, identification of subgroups of DTF patients with similar symptom burden may help to identify patients at risk of poor outcomes and to provide care that meets the patients' individual needs.

Therefore, the aims of this study were as follows: (1) to identify subgroups of DTF patients based on symptom burden using a cluster analysis; (2) to compare symptom burden subgroups on HRQoL and healthcare use (univariate and multivariate).

## 2 METHODS

### 2.1 Study sample and data collection

The sample included DTF patients from the United Kingdom (UK) and the Netherlands (NL) who participated in the QUALIFIED study (The evaluation of HRQoL issues experienced by patients with DTF; registered at clinicaltrials.gov: NCT04289077).^18^ The QUALIFIED study is an international, multicentre, cross‐sectional, observational study among adult patients (≥18 years) with sporadic DTF who were treated in one of the participating centres (UK: one centre; NL: three centres). After obtaining informed consent, patients completed a set of questionnaires including the European Organization for Research and Treatment of Cancer Quality of Life Questionnaire Core 30 (EORTC QLQ‐C30), a DTF‐specific HRQoL questionnaire (DTF‐QoL) and questions related to healthcare utilisation. Questionnaire data were collected via the PROFILES management system; an established international registry for collection of cancer patient‐reported outcomes.^19^ Data collection was conducted between August 2020 and February 2021. Ethical and institutional approval was obtained in each participating centre in the UK and the NL. Further details of the protocol have been published previously.^18^

### 2.2 Study measures

#### 2.2.1 Socio‐demographic and clinical characteristics

Socio‐demographic and clinical data were extracted from the patient‐reported questionnaire and from the electronic patient records (EPR). Comorbidities were assessed using an adapted self‐administered comorbidity questionnaire (SCQ),^20^ which included one question about the presence of comorbidities in the previous 12 months. Additional medical data were obtained from the EPR to ensure correct and detailed reporting.^18^ For the analyses, DTF patients were assigned to one of the following treatment groups: ‘only AS’, ‘only surgery’ and ‘other treatment’. Treatment with non‐steroidal anti‐inflammatory drugs (NSAIDs) or other analgesics was not considered as active treatment.^8^ The ‘other treatment’ group included patients who received systemic therapy (i.e. chemotherapy, hormonal therapy and targeted medical therapy), local therapy (i.e. radiotherapy, isolated limb perfusion, high‐intensity focused ultrasound and cryoablation) or a combination of any form of active treatments.

#### 2.2.2 Health‐related quality of life

The EORTC QLQ‐C30 was used to measure HRQoL.^21^ This 30‐item HRQoL questionnaire consists of five functional scales, a global quality of life (QoL) scale, three symptom scales and several single items assessing common symptoms and perceived financial impact of the disease. The timeframe of the questions is the last week. Each item is scored on a Likert scale ranging from 1, ‘not at all’ to 4, ‘very much’, except for the global QoL scale, which is scored on a seven‐point response scale ranging from 1, ‘very poor’ to 7 ‘excellent’. Scores of all scales and single items are linearly transformed to a score between 0 and 100.^22^ A higher score on the functional scales and global QoL means better functioning and HRQoL, whereas a higher score on the symptom scales means higher symptom burden.

DTF‐specific HRQoL was measured by the DTF‐QoL.^23^ The DTF‐QoL was developed according to the guidelines of the EORTC Quality of Life Group to supplement the EORTC QLQ‐C30 and to assess the DTF‐specific issues patients experience.^23^, ^24^ The questionnaire consists of 96 items divided into three symptom scales, 11 disease impact scales and six single items. The timeframe of the symptom scales is ‘the past week’; disease impact scales and single items have a timeframe ‘since diagnosis’, except for the question on sexual interest, which has a timeframe of 4 weeks. Items are scored on a Likert scale with a range of 1, ‘not at all’ to 4 ‘very much’, with an additional ‘not applicable’ option for certain questions. Scores of the DTF‐QoL scales are calculated according to the EORTC QLQ‐C30 scoring manual for symptom scales/items.^22^ After linear transformation of all scales and single items, scores range from 0 to 100. A higher score indicates a higher level of symptoms or problems.

#### 2.2.3 Healthcare use

In the current study, five items were used to assess healthcare utilisation: (1) How many times have you visited your general practitioner (GP) in the last 12 months? (2) How many times have you seen your GP to discuss your DTF or its effects over the last 12 months? (3) How many times have you visited a specialist for DTF over the last 12 months? These questions could be answered by filling in the number of visits or ‘not applicable’ (item 2 and 3) and were asked in a similar way as by Statistics Netherlands (http://statline.cbs.nl/Statsweb/). The last two questions were as follows: (4) Are you comfortable with the follow‐up schedule with your DTF‐specialist? This question could be answered by ‘Yes’, ‘No, I would like to have more appointments’, ‘No, I would like to have fewer appointments’, ‘No, I don't want any appointments’ or ‘Not applicable’; (5) Have you received any care or support from the following people (during or after treatment)? To answer this question, patients could either choose ‘No’ or ‘Yes’ and then choose multiple additional care services from a list: nurse specialist, peer support from other DTF patients, pain specialist (anaesthetist), psychologist, physiotherapist, company doctor, social worker, dietician, art therapist, sex therapist, pastor, occupational therapist, homoeopathic doctor/alternative medicine practitioner or other.

### 2.3 Statistical analysis

Latent class cluster analysis (LCCA) was conducted to identify clusters of DTF patients based on physical symptom burden, hereafter described as symptom burden. Latent class modelling aims to classify similar objects, with respect to a set of variables, into mutually exclusive groups through a data‐driven and patient‐centred approach.^25^ Variables used to define symptom burden clusters were scores of physical symptom items derived from the EORTC QLQ‐C30 (nausea, dyspnoea, insomnia, appetite loss, constipation, diarrhoea, fatigue, pain) and the DTF‐QoL (unable to lean on tumour site, swelling leg/ankles, stiffness in limbs), dichotomised into ‘no symptoms’ (‘not at all’ i.e. value ‘1’) versus ‘presence of symptoms’ (‘a little’, ‘quite a bit’, ‘very much’ i.e. values ≥2).^26^ Goodness‐of‐fit statistics were used to determine the optimal number of clusters in combination with expert opinion. A detailed description of the LCCA and symptom item selection is presented in Appendix A. Socio‐demographic and clinical characteristics of the identified symptom burden clusters were described using descriptive statistics and compared between clusters with chi‐squared tests for categorical and analysis of variance (ANOVA) for continuous variables.

Because healthcare use was not normally distributed, the three variables describing the number of visits were dichotomised using median split into: visits to GP, 0–1 versus ≥2; visit to GP related to DTF, 0 versus ≥1; visits to DTF‐specialist, 0 versus ≥1. Differences in mean scores of the EORTC QLQ‐C30 functioning scales and mean number of visits to the GP and DTF‐specialist between symptom clusters were analysed using ANOVA with post hoc Bonferroni analysis. Clinically, relevant differences in mean HRQoL scores were determined according to the guidelines of the EORTC Quality of Life Group and divided into small, medium and large clinical differences.^27^

Multivariate linear regression analyses were conducted to investigate the associations between symptom burden and HRQoL. Multivariate logistic regression analyses were conducted to evaluate the relationship between healthcare use as the dependent variable and symptom burden as the independent variable. Other variables included in the multivariate linear and logistic regression analyses were selected a priori: age, sex, relationship status, education level, current employment status, comorbidities, time since diagnosis, received treatment, recurrence and tumour location. LCCA was performed using Latent GOLD 5.2.0 (Statistical Innovations). Further analyses were performed using IBM SPSS Statistics 28.0 (SPSS Inc.). For all analyses, *p*‐values of <0.05 were considered statistically significant.

## 3 RESULTS

### 3.1 Patient characteristics

Two hundred and thirty‐five DTF patients completed the EORTC QLQ‐C30, DTF‐QoL and healthcare utilisation questionnaire (response rate 46%) and were included in the cluster analysis. Socio‐demographic and clinical characteristics of the total study population are shown in Table 1 and described in detail previously.^28^

**TABLE 1:** Desmoid‐type fibromatosis patient characteristics per symptom burden cluster.

	Total study population (*N* = 235), *n* (%)	Cluster 1 (Low), *n* = 57, *n* (%)	Cluster 2 (Intermediate–low pain), *n* = 46, *n* (%)	Cluster 3 (intermediate–high pain), *n* = 59, *n* (%)	Cluster 4 (High), *n* = 73, *n* (%)	*p*‐value
Age at time of questionnaire (years), mean (SD)	47 (14.0)	48 (12.9)	50 (13.7)	43 (12.7)	49 (15.5)	0.053
Age at time of diagnosis (years), mean (SD)	42 (14.4)	42 (13.3)	45 (13.4)	38 (13.1)	42 (16.3)	0.075
Time since diagnosis (years), mean (SD)	5.7 (4.5)	5.5 (3.6)	5.2 (3.5)	5.5 (5.4)	6.3 (4.9)	0.536
Sex						
Female	173 (74)	39 (68)	28 (61)	45 (76)	61 (84)	**0.036**
Relationship status						
Partnered	181 (77)	44 (77)	33 (72)	46 (78)	58 (80)	0.894
Education level						
Low	36 (15)	9 (16)	6 (13)	5 (9)	16 (22)	0.135
Middle	126 (54)	31 (54)	24 (52)	29 (49)	42 (58)	
High	73 (31)	17 (30)	16 (35)	25 (42)	15 (20)	
Current employment status						
Working	155 (66)	44 (77)	31 (67)	39 (66)	41.(56)	0.095
Comorbidities (self‐report)						
None	90 (38)	34 (60)	16 (35)	27 (46)	13 (18)	<0.001
1	74 (32)	17 (30)	14 (30)	23 (39)	20 (27)	
≥2	71 (30)	6 (10)	16 (35)	9 (15)	40 (55)	
Tumour localisation						
Abdominal wall	58 (25)	21 (37)	9 (20)	14 (24)	14 (19)	**<0.001**
Intra‐abdominal	39 (17)	9 (16)	19 (42)	3 (5)	8 (11)	
Head/neck	13 (5)	5 (9)	1 (2)	3 (5)	4 (6)	
Upper extremity/shoulder	29 (12)	6 (10)	2 (4)	10 (17)	11 (15)	
Lower extremity	22 (9)	3 (5)	2 (4)	8 (14)	9 (12)	
Trunk^a^	54 (23)	10 (18)	11 (24)	16 (27)	17 (23)	
Hip/pelvis/gluteal region	20 (9)	3 (5)	2 (4)	5 (8)	10 (14)	
Recurrent disease after surgery (*n* = 98)						
Yes	41 (42)	10 (38)	6 (27)	8 (42)	17 (55)	0.397
Received treatment^b^						
Only active surveillance	87 (37)	27 (47)	15 (33)	26 (44)	19 (26)	**0.005**
Only surgery	64 (27)	17 (30)	20 (44)	9 (15)	18 (25)	
Only systemic therapy	32 (14)	3 (5)	6 (13)	8 (14)	15 (20)	
Only local therapy	8 (3)	1 (2)	3 (6)	2 (3)	2 (3)	
Combination of active treatments	44 (19)	9 (16)	2 (4)	14 (24)	19 (26)	

*Note*: Bold values indicate significant variables (*p* < 0.05).

^a^ Including back, breast, and thoracic wall.

^b^ Systemic therapy: chemotherapy, hormonal therapy, and targeted medical therapy; Local therapy: radiotherapy, isolated limb perfusion, high‐intensity focused ultrasound, cryoablation; Combination of active treatments: different combinations of surgery, systemic therapy, or local therapy.

In the LCCA, the four‐cluster model was found to have the best model fit based on the more liberal goodness‐of‐fit statistics (Akaike's Information Criterion [AIC], Modified AIC; Table S1). Although the three‐cluster model had the best model fit based on the more conservative model statistics (Bayesian Information Criterion, Consistent AIC), the four‐cluster model was found to best describe the variation of symptom burden in DTF patients based on expert opinion, as it identified two distinct subgroups of patients with intermediate levels of physical symptoms but different levels of pain scores.

**FIGURE 1:**
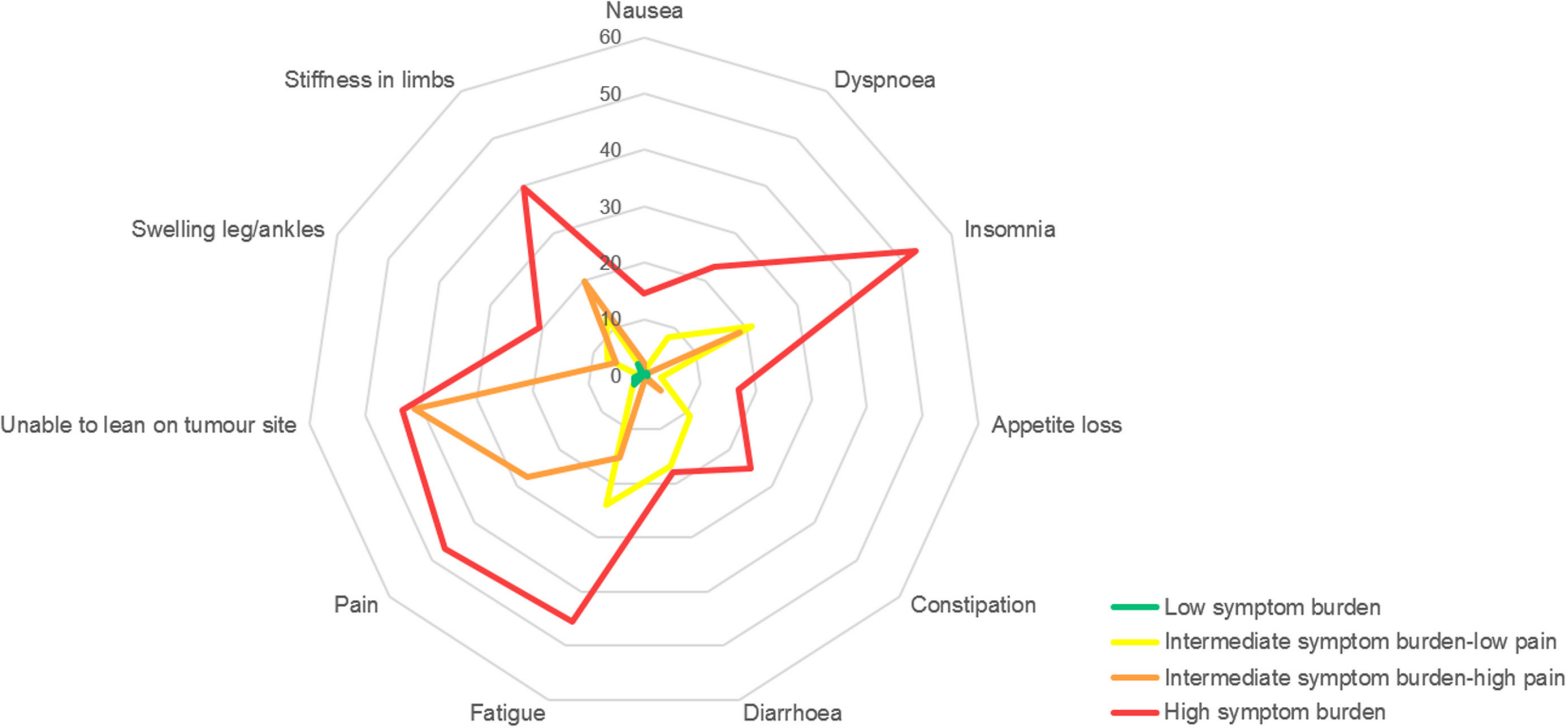
EORTC‐QLQ‐C30 and DTF‐QoL symptom scores per symptom burden cluster using a 0–100 scale with higher scores indicating more complaints. EORTC QLQ‐C30 items: (nausea, dyspnoea, insomnia, appetite loss, constipation, diarrhoea, fatigue, pain); DTF‐QoL items (unable to lean on tumour site, swelling leg/ankles, stiffness in limbs). DTF, desmoid‐type fibromatosis.

Insomnia, unable to lean on tumour site, fatigue and pain were the most frequently reported symptoms for all DTF patients. The four clusters consisted of DTF patients with (1) low symptom burden (*n* = 57;24%), (2) intermediate symptom burden–low pain, that is low scores on the symptoms ‘pain’ and ‘unable to lean on tumour site’, (*n* = 46; 20%), (3) intermediate symptom burden–high pain, that is high scores on ‘pain’ and ‘unable to lean on tumour site’ (*n* = 59; 25%), and (4) high symptom burden (*n* = 73; 31%; Figure 1).

Sex distribution (*p* = 0.036), number of comorbidities (*p* < 0.001), tumour localisation (*p* < 0.001) and received treatments (*p* = 0.005) differed significantly between symptom clusters. The number of DTF patients who were female, had multiple comorbidities and tumours located in the extremities and hip/pelvis/gluteal region was higher in the high symptom burden cluster. The number of patients who received only AS was comparable between the symptom burden clusters.

### 3.2 HRQoL and symptom burden

**FIGURE 2:**
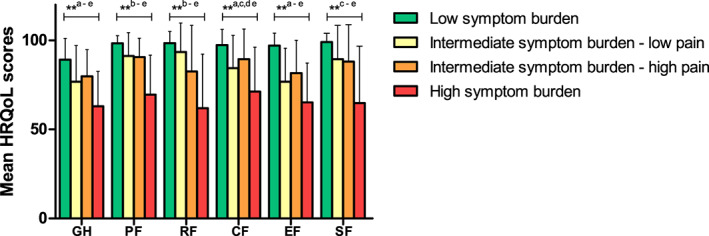
Mean health‐related quality of life (HRQoL) scores of the EORTC QLQ‐C30 global QoL and functional scales by symptom burden cluster with higher scores indicating better HRQoL. **p < 0.01 of ANOVA for differences between clusters. ^a–e^Shows which groups are significantly different according to the Bonferroni post hoc analysis (*p* < 0.05): Low symptom cluster versus: ^a^intermediate–low pain, ^b^intermediate–high pain, ^c^high; High symptom cluster versus: ^d^intermediate–low pain, ^e^intermediate–high pain. HRQoL, health‐related quality of life; GH, global health status; PF, physical functioning; RF, role functioning; CF, cognitive functioning; EF, emotional functioning; SF, social functioning.

DTF patients experiencing low symptom burden reported the highest scores on global QoL and all functioning scales (Figure 2; Table S2). Patients with high symptom burden scored significantly lower on all domains compared to patients with low and intermediate symptom burden. Differences between scores on global QoL and physical, role, cognitive and social functioning of patients in the low and high symptom burden clusters were of large clinical relevance (Table S2).^27^

In the multivariate regression analyses, high symptom burden was independently associated with poorer HRQoL on all domains (Table 2). Intermediate symptom burden, both low and high pain clusters, was negatively associated with global QoL, physical and emotional functioning. Intermediate symptom burden‐low pain was negatively associated with cognitive functioning and intermediate symptom burden‐high pain with role functioning. As for the other socio‐demographic and clinical variables, being employed was associated with better scores on all functioning scales. Having ≥2 comorbidities was only associated with worse global QoL. Longer time since diagnosis was associated with better global QoL, role, cognitive and social functioning. Treatment other than only AS and surgery was associated with worse global QoL, role, emotional and social functioning.

**TABLE 2:** Standardised betas of multivariate linear regression analyses evaluating the associations between symptom burden and HRQoL, adjusted for socio‐demographic and clinical characteristics.

	EORTC QLQ‐C30 Functioning scales^a^					
	Global QoL	Physical functioning	Role functioning	Cognitive functioning	Emotional functioning	Social functioning
Symptom burden						
Cluster 1 (Low)	Ref.	Ref.	Ref.	Ref.	Ref.	Ref.
Cluster 2 (Intermediate–low pain)	−0.21**	−0.15*	−0.11	−0.26**	−0.41**	−0.12
Cluster 3 (Intermediate–high pain)	−0.16*	−0.15*	−0.22**	−0.14	−0.25**	−0.13
Cluster 4 (High)	−0.46**	−0.60**	−0.56**	−0.50**	−0.61**	−0.49**
Age	0.07	−0.06	0.11	−0.02	0.15*	0.11
Sex	0.03	0.15**	0.09	−0.01	0.03	0.04
Relationship status	0.07	0.10	0.11*	0.07	0.09	0.10
Education level						
Low	Ref	Ref	Ref	Ref	Ref	Ref
Medium	−0.03	−0.04	−0.07	−0.03	−0.08	−0.13
High	0.18*	0.04	0.08	0.03	0.004	0.04
Employment status	0.05	0.16**	0.16**	0.13*	0.13*	0.13*
Comorbidity						
None	Ref	Ref	Ref	Ref	Ref	Ref
1	−0.11	0.05	0.07	−0.05	−0.03	0.02
≥2	−0.24**	−0.07	0.02	−0.08	−0.03	−0.09
Time since diagnosis	0.17**	0.09	0.25**	0.14*	0.10	0.16**
Treatment received^b^						
Only active surveillance	Ref	Ref	Ref	Ref	Ref	Ref
Only surgery	−0.04	−0.07	−0.002	−0.07	−0.07	−0.01
Other treatment	−0.19**	−0.09	−0.23**	−0.10	−0.24**	−0.23**
Recurrence	0.07	−0.03	−0.03	−0.05	0.001	−0.06
Tumour						
Abdominal wall	Ref	Ref	Ref	Ref	Ref	Ref
Intra‐abdominal	0.09	0.11	0.12	0.21**	0.21**	0.05
Upper extremity	0.05	0.07	0.09	0.05	0.05	−0.01
Lower extremity	0.01	−0.02	0.03	0.04	0.08	−0.01
Head/neck	0.11	0.01	−0.04	0.09	0.02	0.07
Trunk	0.09	0.06	0.14*	0.15*	0.07	0.07
Hip/pelvis/gluteal region	0.03	−0.04	0.03	0.06	0.06	0.05

*Note*: Age at time of diagnosis and time since diagnosis were continuous variables. The categorical variables symptom burden, education level, comorbidity, treatment received and tumour anatomic location, had >2 categories and were transformed into dummy variables, with, respectively, low symptom burden, low, none, only AS and abdominal wall as the reference groups. Other categorical variables were as follows: sex: female vs. male; relationship status: not partnered vs. partnered; current employment status: not working vs. working; recurrent disease after surgery: not recurrent vs. recurrent.

^a^ Higher score indicates better functioning.

^b^ Active surveillance only and surgery only: including patients who received analgesics. Other treatment, including patients who received only systemic therapy (i.e., chemotherapy, hormonal therapy, targeted medical therapy) or local therapy (i.e., radiotherapy, isolated limb perfusion, high‐intensity‐focused ultrasound, cryoablation) or a combination of any form of active treatments.

* *p* < 0.05;

** *p* < 0.01.

### 3.3 Healthcare use and symptom burden

**FIGURE 3:**
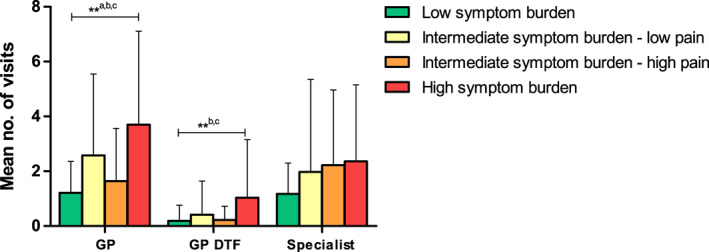
Mean number of visits of DTF patients to the general practitioner in general (GP), in relation to their DTF (GP DTF), and specialist for DTF (specialist) by symptom burden cluster. **p < 0.01 of ANOVA for differences between clusters. ^a–c^Shows which groups are significantly different according to the Bonferroni post hoc analysis (*p* < 0.05): Low symptom cluster versus: ^a^intermediate–low pain; High symptom cluster versus: ^b^low, ^c^intermediate–high pain. no, number; GP, general practitioner; DTF, desmoid‐type fibromatosis.

DTF patients who experienced high symptom burden reported significantly more general and DTF‐related visits to the GP than patients who experienced low or intermediate symptom burden–high pain (Figure 3). No significant differences in the number of DTF‐specialist visits were found between the four symptom burden clusters. Whether patients felt comfortable with their follow‐up schedule with their DTF‐specialist was significantly different between the symptom burden clusters (*p* = 0.048), with patients in the high symptom burden cluster more often preferring more appointments with their DTF‐specialist (Table S3). DTF patients in the intermediate symptom burden–high pain and high symptom burden clusters were more frequently receiving additional care, mostly from a physiotherapist or psychologist (Table S4).

Multivariate logistic regression analyses showed that DTF patients visiting their GP ≥2 were more likely to experience high symptom burden and to report ≥1 comorbidities than patients who reported 0–1 visit to their GP (Table 3). DTF patients who visited their GP ≥1 for their DTF were more likely to be female, to have high symptom burden and to have a recent diagnosis than patients who did not visit their GP to discuss their DTF. Patients visiting their DTF‐specialist ≥1 were more likely to have a recent diagnosis, to receive treatments other than only AS or surgery and to have recurrent disease. No association was seen between symptom burden and the number of visits to a DTF‐specialist.

**TABLE 3:** Multivariate logistic regression analyses evaluating associations between symptom burden and healthcare use, adjusted for socio‐demographic and clinical characteristics (odds ratio and 95% confidence interval).

	General practitioner visits past 12 months		DTF‐specialist visits past 12 months
	≥2 general visits	≥1 DTF‐related visit	≥1 DTF‐related visit
Symptom burden			
Cluster 1 (Low)	Ref.	Ref.	Ref.
Cluster 2 (Intermediate–low pain)	2.44 (0.94–6.31)	1.38 (0.25–7.52)	1.10 (0.41–3.01)
Cluster 3 (Intermediate–high pain)	0.90 (0.39–2.11)	2.85 (0.71–11.48)	0.87 (0.33–2.34)
Cluster 4 (High)	**2.71 (1.08–6.76)**	**10.35 (2.32–46.16)**	1.39 (0.48–4.06)
Age	1.00 (0.97–1.03)	0.97 (0.94–1.00)	1.00 (0.97–1.03)
Sex	0.67 (0.31–1.42)	**5.46 (1.92–15.51)**	0.98 (0.42–2.27)
Relationship status	1.14 (0.54–2.39)	0.81 (0.28–2.32)	0.87 (0.35–2.14)
Education level			
Low	Ref	Ref	Ref
Medium	1.21 (0.47–3.10)	1.07 (0.27–4.25)	0.75 (0.25–2.26)
High	0.96 (0.33–2.82)	2.65 (0.59–11.86)	1.01 (0.29–3.48)
Employment status	0.95 (0.45–2.02)	1.16 (0.44–3.03)	0.54 (0.22–1.30)
Comorbidity			
None	Ref	Ref	Ref
1	**3.23 (1.56–6.68)**	0.72 (0.24–2.15)	0.78 (0.34–1.78)
≥2	**5.06 (2.15–11.92)**	2.31 (0.76–7.01)	1.36 (0.49–3.76)
Time since diagnosis	0.98 (0.91–1.06)	**0.85 (0.76–0.95)**	**0.77 (0.69–0.86)**
Treatment received^a^			
Only active surveillance	Ref	Ref	Ref
Only surgery	1.06 (0.45–2.49)	0.83 (0.24–2.88)	0.60 (0.25–1.44)
Other treatment	1.15 (0.50–2.64)	1.44 (0.48–4.37)	**3.28 (1.14–9.46)**
Recurrence	1.54 (0.60–3.96)	2.59 (0.81–8.28)	**10.76 (2.60–44.47)**
Tumour location			
Abdominal wall	Ref.	Ref.	Ref.
Intra‐abdominal	0.41 (0.15–1.16)	0.68 (0.15–3.22)	0.51 (0.18–1.50)
Upper extremity	1.85 (0.56–6.10)	0.33 (0.07–1.62)	4.36 (0.88–21.52)
Lower extremity	0.36 (0.10–1.29)	0.37 (0.08–1.82)	1.33 (0.32–5.47)
Head/neck	0.40 (0.09–1.71)	0.96 (0.14–6.63)	0.65 (0.12–3.47)
Trunk	0.57 (0.24–1.40)	0.36 (0.09–1.38)	0.95 (0.37–2.44)
Hip/pelvis/gluteal region	2.23 (0.60–8.39)	1.31 (0.29–5.89)	9.38 (1.01–87.74)

*Note*: Age at time of diagnosis and time since diagnosis were continuous variables. The categorical variables symptom burden, education level, comorbidity, treatment received and tumour anatomic location, had >2 categories with, respectively, low symptom burden, low, none, only AS and abdominal wall as the reference groups. Other categorical variables were as follows: sex: female versus male; relationship status: not partnered versus partnered; current employment status: not working versus working; recurrent disease after surgery: not recurrent versus recurrent. Bold values indicate significant variables (*p* < 0.05).

^a^ Active surveillance only and surgery only: including patients who received analgesics. Other treatment, including patients who received only systemic therapy (i.e., chemotherapy, hormonal therapy, targeted medical therapy) or local therapy (i.e., radiotherapy, isolated limb perfusion, high‐intensity‐focused ultrasound, cryoablation) or a combination of any form of active treatments.

## 4 DISCUSSION

This study demonstrated that symptom burden varied widely between DTF patients, with a considerable number of patients being highly symptomatic. Four subgroups of DTF patients were identified based on their symptom burden: those with low symptom burden, those with intermediate symptom burden and low pain, those with intermediate symptom burden and high pain, and those with high symptom burden. High symptom burden was associated with poor functioning and higher healthcare use.

Pain, unable to lean on tumour site, fatigue and insomnia were the most prevalent symptoms in our study population. These physical symptoms are consistent with those observed in previous studies in DTF patients and highlight the functional burden DTF patients can experience.^9^, ^10^, ^11^, ^29^, ^30^ Interventions targeting physical symptoms could improve HRQoL of DTF patients, but this requires a careful look at different subgroups with the same degree of symptom burden to know what to intervene on. To the best of our knowledge, this is the first study that identified relatively distinct subgroups of patients based on their level of symptom burden in a heterogeneous population of DTF patients using LCCA.

In studies with cancer patients, the number and type of symptom clusters varied, depending on study design, type of cancer and type of symptoms used. Most studies identified at least an ‘all low’ and ‘all high’ symptom subgroup, with worse HRQoL outcomes for patients with high symptom burden.^12^, ^13^, ^14^, ^31^, ^32^ Consistent with these results, our study also identified subgroups of DTF patients with low or high scores on all symptoms. Additionally, two distinct subgroups of patients with both intermediate symptom burden but with either more prominent symptoms due to distress or physical symptoms were identified. Differences in HRQoL outcomes between these four symptom clusters emphasise the need to distinguish these subgroups to provide DTF patients with appropriate supportive care. DTF patients with high symptom burden reported remarkably low HRQoL scores compared to patients with low and intermediate symptom burden, independent of other socio‐demographic and clinical variables and with differences of large clinical relevance between patients in the low and high symptom clusters. Intermediate symptom burden also resulted in lower HRQoL scores. Differences between the two intermediate clusters were seen in role and cognitive functioning. Patients with intermediate symptom burden‐low pain appear to have more difficulty concentrating and remembering, possibly due to experiencing more fatigue and insomnia. This pattern of symptoms may be the result of distress due to DTF itself, or due to the use of opioids. These medications may result in patients reporting less pain but experiencing other symptoms, thus falling into the intermediate symptom burden‐low pain cluster. On the contrary, DTF patients with high pain scores seem to be more restricted in daily activities, possibly due to functional limitations caused by pain from the DTF tumour. This supports the methodological decision to distinguish between these two intermediate clusters as their symptoms lead to different problems requiring different supportive care.

Previous studies have shown that cancer survivors reported more healthcare use than the general population and that the number of visits is influenced by several factors, such as illness perception, number of follow‐up visits, and physical and psychological complaints.^33^, ^34^, ^35^ Our study is one of the first to evaluate healthcare use by DTF patients. No differences in healthcare use were found between patients from the UK and NL. High symptom burden was independently associated with general and DTF‐related visits of DTF patients to their GP. Moreover, patients with high symptom burden reported more frequent use of additional care services, mainly a nurse specialist, physiotherapist, psychologist or pain specialist. In line with previous studies in cancer survivors, the presence of comorbidities was related to general visits to the GP by DTF patients.^33^, ^36^ Female DTF patients were more likely to visit their GP to discuss their DTF. Possible explanations mentioned in previous studies for why women are more likely to use health services include differences in health perception and social roles.^37^, ^38^, ^39^ Further research should be conducted to understand the more frequent use of health care in female DTF patients.

DTF patients with intermediate symptom burden‐low pain reported more visits to their GP compared to patients with intermediate symptom burden‐high pain. This may be due to the higher number of patients with comorbidities in the intermediate symptom burden‐low pain cluster. This is supported by our multivariate analysis in which intermediate symptom burden‐low pain was not associated with the number of GP visits, but the presence of comorbidities was. Another explanation might be that DTF patients with intermediate symptom burden‐low pain experienced more general complaints of fatigue, constipation and diarrhoea, which, unlike the symptoms related to pain and unable to lean on tumour site, are less likely to be attributed to their DTF by patients. Finally, a higher percentage of patients with intermediate symptom burden‐high pain reported the use of additional care services, for example a physiotherapist, which may also have reduced their visits to the GP.

Symptom burden was not associated with the number of visits to a DTF‐specialist. This may suggest that the threshold for patients to visit a medical specialist is too high. DTF patients with high symptom burden did report more DTF‐related visits to their GP and indicated that they would like to have more appointments with their DTF‐specialist. However, one could question whether more visits to a DTF‐specialist would reduce symptom burden, or whether DTF‐specialists should instead refer patients more often to additional care services. Treatments other than AS or surgery only, recurrent disease and a shorter time since diagnosis were associated with more visits to a DTF‐specialist. This may be because other treatments, such as systemic therapy, require more frequent appointments with DTF‐specialists. Recurrent disease may require additional treatment, or may result in more concerns about their disease, resulting in more appointments. DTF patients with shorter time since diagnosis visited their DTF‐specialist more frequently, reflecting follow‐up care according to international guidelines and an increase in understanding of this unusual condition in due course.^8^ Given the small number of patients in some subgroups, resulting in large confidence intervals, the findings on the associations between healthcare use and socio‐demographic and clinical variables should be replicated in future longitudinal studies with larger numbers.

A limitation of this study was that other factors that may influence physical symptom reporting, HRQoL and healthcare utilisation, such as illness perception, depression and anxiety, were not included.^15^, ^34^, ^40^, ^41^ Despite the inclusion of patients from two countries, the type of healthcare system was not included because previous studies have found that patients from the UK experience higher symptom burden which could have led to biased results.^11^, ^42^ There may be selection bias, as it is unknown whether DTF patients did not respond or participate, due to either the absence of symptoms or poor health.^43^ The non‐responder analysis did not reveal any differences; however, clinical characteristics were unavailable for these patients.^28^ Type of pain and type of pain medication were not included in this study, although these may affect the level of physical symptom burden. In addition, healthcare utilisation was based on self‐report which may have suffered from recall bias. Finally, the EORTC‐QLQ‐C30 and DTF‐QoL collect information on both symptoms and HRQoL, which may lead to common method bias. Common method bias can occur when both the independent and dependent variables are collected using the same method, potentially resulting in false correlations.^44^ Other limitations of the QUALIFIED study, including the cross‐sectional study design, have been described in detail previously.^28^ Despite these limitations, strengths could also be identified. The study included a large study population of DTF patients, and both generic and disease‐specific questionnaires were used. Furthermore, this is the first study identifying symptom burden clusters in DTF using LCCA and describing healthcare use of DTF patients from the UK and NL.

### 4.1 Clinical implications

Our results highlight the need to recognise highly symptomatic DTF patients in clinical practice. It is important to be aware that our study population included a relatively high percentage of patients who underwent surgical resection and a low percentage of patients who received targeted medical therapy (i.e. tyrosine kinase and gamma‐secretase inhibitors). Our results should therefore be interpreted in the changing landscape of treatment options for DTF.^45^, ^46^, ^47^ DTF patients in the intermediate symptom burden‐high pain and high symptom burden cluster, who were more often female and had tumours located in the extremities and hip/pelvic/gluteal region, may need more frequent follow‐up visits to their medical specialist, more appropriate supportive care, for example by a pain specialist, or seem to be the good candidates for gamma secretase inhibitors.^45^, ^46^, ^47^ On the contrary, patients in the low symptom burden cluster, in whom the tumours were more frequently located in the abdominal wall, may need less frequent follow‐up visits. The number of patients who received only AS was relatively similar between tumour locations (data not shown). DTF patients in the low symptom burden cluster relatively often received only AS. However, a significant proportion of patients in the intermediate symptom burden‐high pain and high symptom burden cluster also received only AS. Therefore, it is important to monitor pain in patients who are under AS to provide adequate pain management to keep symptom burden as low as possible. DTF patients who remain highly symptomatic may need to change to active treatment. Even though patients in the more symptomatic clusters made more frequent use of primary care and additional support, this care is not yet sufficiently tailored to the needs of the patients because they remain highly symptomatic with poorer HRQoL. Symptom monitoring during routine clinical care using patient‐reported outcomes (PROs) have been shown to alert clinicians to intensify symptom management and to improve symptom control, resulting in better HRQoL and fewer hospital admissions in cancer patients.^48^ Future research could assess the use of electronic self‐report of symptoms using generic and DTF‐specific PROs in DTF patients, which could provide valuable information on symptom burden, leading to appropriate supportive care.

## 5 CONCLUSION

Four relatively distinct subgroups of DTF patients were identified based on their level of symptom burden. A higher symptom burden negatively affects HRQoL outcomes and results in increased healthcare use. A better understanding of which DTF patients experience low or high symptom burden can help to identify patients at risk of poorer outcomes and to tailor active treatment and supportive care to the individual needs of DTF patients.

## AUTHOR CONTRIBUTIONS

**Anne‐Rose W. Schut:** Conceptualization (lead); data curation (lead); formal analysis (lead); methodology (equal); project administration (lead); resources (equal); validation (lead); visualization (lead); writing – original draft (lead); writing – review and editing (lead). **Leanne E de Bruin:** Formal analysis (lead); methodology (equal); validation (equal); writing – review and editing (equal). **Belle H de Rooij:** Formal analysis (equal); methodology (equal); writing – review and editing (supporting). **Emma Lidington:** Data curation (equal); resources (lead); writing – review and editing (supporting). **Milea J.M. Timbergen:** Funding acquisition (lead); project administration (equal); resources (equal); writing – review and editing (supporting). **Winette TA van der Graaf:** Resources (supporting); writing – review and editing (supporting). **Winan J. van Houdt:** Resources (supporting); writing – review and editing (supporting). **Johannes J. Bonenkamp:** Resources (equal); writing – review and editing (supporting). **Robin L. Jones:** Resources (supporting); writing – review and editing (supporting). **Dirk J. Grünhagen:** Conceptualization (equal); resources (equal); writing – review and editing (equal). **Stefan Sleijfer:** Resources (supporting); writing – review and editing (equal). **Spyridon Gennatas:** Data curation (equal); resources (equal); writing – review and editing (supporting). **Cornelis Verhoef:** Conceptualization (equal); funding acquisition (equal); resources (equal); writing – review and editing (equal). **Olga Husson:** Conceptualization (lead); data curation (equal); formal analysis (lead); funding acquisition (equal); methodology (lead); project administration (equal); resources (equal); supervision (lead); validation (equal); visualization (lead); writing – review and editing (equal).

## FUNDING INFORMATION

This research was funded by Stichting Coolsingel, Rotterdam, the Netherlands (grant number 566), the National Institute for Health Research (NIHR) Biomedical Research Centre at the Royal Marsden NHS Foundation Trust and the Institute of Cancer Research, London (B038). O.H. is supported by a grant from the Netherlands Organisation for Scientific Research (VIDI198.007).

## CONFLICT OF INTEREST STATEMENT

R.L.J. has received research grants from MSD and GSK and is a consultant for Adaptimmune, Athenex, Bayer, Boehringer Ingelheim, Blueprint, Clinigen, Eisai, Epizyme, Daichii, Decipheara, Immunedesign, Lilly, Merck, Pharmacar, Springworks, Tracon and UptoDate. W.T.A.v.d.G. has received a research grant from Lilly, and advisory board fees from Bayer, Springworks and PTC therapeutics; all fees to the institute. The funders had no role in the design of the study; in the collection, analyses or interpretation of data; in the writing of the manuscript, or in the decision to publish the results.

## Supporting information


Appendix S1.

## Data Availability

The datasets used and/or analysed during the current study are available from the corresponding author on reasonable request.
