# Supplementary material for: Physical symptom burden in patients with desmoid‐type fibromatosis and its impact on health‐related quality of life and healthcare use
Source: Cancer Med. 2023 Apr 29;12(12):13661–74. doi: 10.1002/cam4.5985 (PMC10315709; doi:10.1002/cam4.5985)
Supplement: Supplementary file 1 — Appendix S1. [file CAM4-12-13661-s001.pdf]

**Appendix A – Supplementary Materials**

**Supplementary Methods**

*Statistical analysis*

Latent class cluster analysis was conducted to identify clusters of desmoid-type fibromatosis (DTF) patients based on physical symptom burden, hereafter described as symptom burden. Latent class modeling aims to classify similar objects, with respect to a set of variables, into mutually exclusive groups through a data-driven and patient-centered approach (1). Variables used to define symptom clusters in this study were dichotomous scores of physical symptom items derived from the European Organization for Research and Treatment of Cancer Quality of Life Questionnaire Core 30 (EORTC QLQ-C30) and the DTF-specific Health-Related Quality of Life questionnaire (DTF-QoL). The EORTC QLQ-C30 symptom scales 'pain' and 'fatigue' were incorporated as scale scores to prevent correlation between the scores of the single items. Physical items from the impact scales of the DTF-QoL were not included due to the different time frames. Additionally, symptom items reported by <10% of the study population were not included in the cluster analysis. Eight symptoms derived from the EORTC QLQ-C30 (nausea, dyspnea, insomnia, appetite loss, constipation, diarrhea, fatigue, pain) and three symptoms from the DTF-QoL (unable to lean on tumor site, swelling leg/ankles, stiffness in limbs) were used to define the symptom burden clusters. Because symptom scores were not normally distributed, all symptom scores were dichotomized into 'no symptoms' ('not at all' i.e. value '1') versus 'presence of symptoms' ('a little', 'quite a bit', 'very much' i.e. values ≥2) (2). Goodness-of-fit statistics (lowest values of: Log-likelihood [LL] of Bayes Information Criterion [BIC], LL of Akaike's Information Criterion [AIC], LL of Consistent Akaike's Information Criterion [CAIC]) were used to determine the optimal number of clusters in combination with expert opinion. Bivariate residuals were assessed to check if the local independency assumption was met (values <3). When bivariate residuals remain high with increasing number of classes in the model, the local independency assumption was relaxed (1). Latent class cluster analysis was performed using Latent GOLD 5.2.0 (Statistical Innovations, Belmont, MA, USA).

**References**

1. Vermunt JK, Magidson J. Latent GOLD 4.0 User's Guide. Belmont, Massachusetts: Statistical Innovations Inc; 2005.
2. Weidema ME, Husson O, van der Graaf WTA, Leonard H, de Rooij BH, Hartle DeYoung L, et al. Health-related quality of life and symptom burden of epithelioid hemangioendothelioma patients: a global patient-driven Facebook study in a very rare malignancy. Acta Oncol. 2020;59(8):975-82.

## Supplementary Tables

**Supplementary Table S1.** Fit statistics of latent class analysis

| Model | LL | BIC(LL) | AIC(LL) | AIC3(LL) | CAIC(LL) | Npar | $L^2$ | df | *p*-value | Class.Err. |
|---|---|---|---|---|---|---|---|---|---|---|
| 1-Cluster | -1464,2129 | 2988,4812 | 2950,4258 | 2961,4258 | 2999,4812 | 11 | 891,8955 | 224 | 1,9e-80 | 0,0000 |
| 2-Cluster | -1272,2423 | 2670,0550 | 2590,4846 | 2613,4846 | 2693,0550 | 23 | 507,9543 | 212 | 2,5e-26 | 0,0548 |
| 3-Cluster | -1237,9661 | **2667,0176** | 2545,9321 | 2580,9321 | **2702,0176** | 35 | 439,4019 | 200 | 5,2e-20 | 0,0973 |
| 4-Cluster | -1213,8560 | 2684,3125 | **2521,7120** | **2568,7120** | 2731,3125 | 47 | 391,1817 | 188 | 2,3e-16 | 0,1091 |
| 5-Cluster | -1202,0502 | 2726,2160 | 2522,1005 | 2581,1005 | 2785,2160 | 59 | 367,5702 | 176 | 1,3e-15 | 0,1031 |
| 6-Cluster | -1192,8178 | 2773,2661 | 2527,6356 | 2598,6356 | 2844,2661 | 71 | 349,1053 | 164 | 2,0e-15 | 0,1291 |
| | | | | | | | | | | |
| 4-Cluster* | -1209,9740 | 2682,0081 | 2515,9480 | 2563,9480 | 2730,0081 | 48 | 383,4178 | 187 | 1,2e-15 | 0,1103 |

Model with the lowest model statistic values was deemed the model with the best fit. Lowest values are in bold.
*Final cluster model. The local independency assumption was relaxed for appetite loss and nausea.
Abbreviations: LL: Log-likelihood; BIC(LL): Bayesian Information Criterion (based on LL); AIC(LL):Akaike's Information Criterion (based on LL); AIC3(LL): Modified AIC (based on LL); CAIC (LL): Consistent Akaike Information Criterion (based on LL); Npar: Number of parameters; L2: L-squared; df: degrees of freedom; Class. Err.: Classification errors.

**Supplementary Table S2.** Differences in mean scores of the EORTC QLQ-C30 global health and functional scales by symptom burden cluster

| | Mean (SD) | | | | |
|---|---|---|---|---|---|
| | Cluster 1<br>(Low)<br>n = 57<br>(24%) | Cluster 2<br>(Intermediate – low pain)<br>n = 46<br>(20%) | Cluster 3<br>(Intermediate – high pain)<br>n = 59<br>(25%) | Cluster 4<br>(High)<br>n = 73<br>(31%) | *p*-value |
| Global QoL | 89.2 (11.9) | 76.8 (20.3) | 79.9 (14.9) | 63.0 (19.6) | <0.001[a*,b#,c†,d*,e†] |
| Physical functioning | 98.4 (4.2) | 91.2 (13.2) | 90.6 (10.6) | 69.6 (22.1) | <0.001[b#,c†,d*,e*] |
| Role functioning | 98.5 (6.5) | 93.5 (16.3) | 82.5 (26.0) | 61.9 (30.4) | <0.001[b#,c†,d†,e*] |
| Cognitive functioning | 97.4 (8.8) | 84.4 (18.4) | 89.5 (16.9) | 71.2 (25.0) | <0.001[a*,c†,d*,e†] |
| Emotional functioning | 97.1 (7.0) | 76.8 (18.8) | 81.6 (18.4) | 65.2 (22.1) | <0.001[a,b,c,d,e] |
| Social functioning | 99.1 (4.9) | 89.5 (19.0) | 88.1 (20.8) | 64.8 (32.0) | <0.001[c†,d†,e†] |

[a] 1 (Low) vs 2 (Intermediate – low pain)
[b] 1 (Low) vs 3 (Intermediate – high pain)
[c] 1 (Low) vs 4 (High)
[d] 2 (Intermediate – low pain) vs 4 (High)
[e] 3 (Intermediate – high pain) vs 4 (High)
# Small clinical difference, * Medium clinical difference, † Large clinical difference
Abbreviations: EORTC QLQ-C30, European Organization for Research and Treatment of Cancer Quality of Life Questionnaire Core 30; SD, standard deviation; QoL, quality of life.

**Supplementary Table S3.** Distribution of answers to the question whether patients were comfortable with their follow-up schedule to their desmoid-type fibromatosis specialist by symptom burden cluster (n [%])

| | Cluster 1 (Low) n = 57 (24%) | Cluster 2 (Intermediate – low pain) n = 46 (20%) | Cluster 3 (Intermediate – high pain) n = 59 (25%) | Cluster 4 (High) n = 73 (31%) |
|---|---|---|---|---|
| Not applicable | 13 (23) | 6 (13) | 5 (8) | 3 (4) |
| No, I don't want any appointments | 0 (0) | 0 (0) | 1 (2) | 1 (1) |
| No, I would like to have more appointments | 3 (5) | 3 (7) | 6 (10) | 12 (17) |
| Yes | 41 (72) | 37 (90) | 47 (80) | 57 (78) |

Zero patients selected the answer option 'No, I would like to have fewer appointments'

**Supplementary Table S4.** Type of health care use of desmoid-type fibromatosis patients by symptom burden cluster (n [%])

| | Cluster 1 (Low) n = 57 (24%) | Cluster 2 (Intermediate – low pain) n = 46 (20%) | Cluster 3 (Intermediate – high pain) n = 59 (25%) | Cluster 4 (High) n = 73 (31%) |
|---|---|---|---|---|
| Sarcoma nurse specialist | **15 (26)** | **13 (28)** | **9 (15)** | **23 (32)** |
| Physiotherapist | **7 (12)** | **7 (15)** | **19 (32)** | **23 (32)** |
| Psychologist | 2 (4) | 3 (7) | **11 (19)** | **16 (22)** |
| Pain specialist (anaesthetist) | 2 (4) | **4 (9)** | 6 (10) | **13 (18)** |
| Peer support other DTF patients | **3 (5)** | 3 (7) | **10 (17)** | 11 (15) |
| Company doctor | **4 (7)** | **4 (9)** | 6 (10) | 3 (4) |
| Social worker | 0 (0) | 2 (4) | 2 (3) | 3 (4) |
| Dietician | 0 (0) | 2 (4) | 1 (2) | 6 (8) |
| Occupational therapist | 0 (0) | 0 (0) | 2 (3) | 9 (12) |
| Homeopathic doctor / alternative medicine practitioner | 1 (2) | 2 (4) | 2 (3) | 6 (8) |
| Pastor | 0 (0) | 0 (0) | 0 (0) | 1 (1) |
| Other[a] | 6 (10) | 8 (17) | 4 (7) | 12 (16) |

Bold values indicate the five most frequent additional care services of that particular cluster.
[a] Including medical specialist, family/friends, lymph oedema nurse, stoma nurse, private clinic, osteopath, sport masseuse, guru, shop manager.
